# Gender Differences in the Relationship Between Fatigue, Different Types of Physical Activity, Postural Changes, and Sleep Quality in University Students—Part II Analyses from a Cross-Sectional Study

**DOI:** 10.3390/jfmk10030307

**Published:** 2025-08-08

**Authors:** Verner Marijančić, Silvije Šegulja, Mirela Vučković, Ivana Sović, Stanislav Peharec, Tanja Grubić Kezele, Gordana Starčević-Klasan

**Affiliations:** 1 Department of Physiotherapy, Faculty of Health Studies, University of Rijeka, 51000 Rijeka, Croatia; verner.marijancic@uniri.hr (V.M.); mirela.vuckovic@fzsri.uniri.hr (M.V.); stanislav.peharec@fzsri.uniri.hr (S.P.); 2 Department of Clinical Sciences I, Faculty of Health Studies, University of Rijeka, 51000 Rijeka, Croatia; silvije.segulja@fzsri.uniri.hr; 3 Department of Basic Medical Science, Faculty of Health Studies, University of Rijeka, 51000 Rijeka, Croatia; ivana.sovic@fzsri.uniri.hr; 4 Department of Physiology, Immunology and Pathophysiology, Faculty of Medicine, University of Rijeka, 51000 Rijeka, Croatia; 5 Department of Clinical Microbiology, Clinical Hospital Rijeka, 51000 Rijeka, Croatia

**Keywords:** fatigue, lumbar pain, physical activity, posture, sedentary behavior, sleep quality, trunk extensor endurance, trunk flexor endurance, university students

## Abstract

**Background:** Fatigue can be a useful tool to understand the effects of physical activity (PA) and sedentary behavior on musculoskeletal health in university students. **Methods:** The aim of this cross-sectional study was to examine gender differences in the relationships between fatigue and specific types and levels of PA, posture, sleep quality (SQ), and non-specific low back pain (NS-LBP) in young adult university students aged 18–25 years. A total of 180 students completed all required tests. **Results:** Female students had higher total fatigue as they generally engaged in more PA in contrast to male students, who had higher total fatigue when they engaged in less moderate and less vigorous PA. With increasing sedentary behavior, overall fatigue was pronounced in both sexes, although female students spent significantly more time sitting. Poorer SQ correlated with NS-LBP and higher levels of sleep-related fatigue in female students. Males with pronounced hypekyphosis and females with pronounced lordosis were more fatigued. In addition, fatigue was more pronounced in female students with a higher extensor/flexor ratio, suggesting that trunk extensors are more fatigued due to the need to maintain lumbar spine stability. **Conclusions:** Our findings suggest that the choice of PA should be gender-specific to prevent chronic musculoskeletal disorders and fatigue in young adult university students.

## 1. Introduction

Low levels of physical activity (PA) and sedentary behavior in young people have become the most important public health problems of the 21st century [1,2]. This is because they cause the risk of developing a variety of chronic diseases, such as cardiovascular diseases, diabetes, obesity, cancer, and an increased risk of all-cause mortality [3,4]. In addition, the majority of adolescents and adults do not achieve the levels of PA recommended by the WHO [4,5]. University students represent a particularly vulnerable population group, as they spend more and more time sitting during their studies, which poses a major risk to their musculoskeletal health and posture [6,7,8,9]. Nevertheless, posture and muscle problems, back pain, and fatigue are common consequences of prolonged sitting [10]. Moreover, fatigue can be a useful tool to understand the lifestyle habits of young adults [11]. Fatigue is often associated with a lack of physical and mental strength and a decline in motivation [12], which could hinder the healthy growth of young people. Indeed, if posture is not maintained correctly, areas not intended to support the spine are stressed, and the core of the body fatigues quickly, leading to postural fatigue. In addition, the stress poor posture places on parts of the spine can promote other negative effects, including muscle weakness and discomfort when sitting [13,14].

Furthermore, academic responsibilities during college provide greater academic pressure that leads to mental fatigue [15], i.e., a psychobiological condition caused by prolonged periods of demanding cognitive activity. For example, university students often suffer from academic fatigue because they have to endure hours of classes, exams, competitions, and schoolwork [16]. In addition, mental fatigue also limits exercise tolerance [17] and impairs the duration and intensity of physical tasks and endurance performance in healthy young people. The impairment of endurance performance due to mental fatigue is mediated by a higher than normal perception of effort [18]. Mental fatigue can also impair sport-specific psychomotor performance [19]. In addition, women show greater resilience when faced with fatiguing mental tasks [20], so they should exhibit less academic fatigue during demanding cognitive activities during their studies. This finding shows that gender appears to influence the relationship between fatigue state and brain activity. Many studies have shown that fatigue is associated with physical activity [11,21,22,23]. A low level of exercise is often associated with a high level of physical fatigue [21,22]. Young men usually engage in higher levels of PA than women [9,24], which could be a reason why women experience more fatigue [25]. However, this may not be entirely true, as there is no definitive standardized definition of fatigue, especially between genders. Nonetheless, sport overuse can also have negative effects on central nervous system drive or cause physical fatigue [26]. Furthermore, when comparing physical fatigue between genders, it has been shown that women exhibit different muscle activation patterns in certain exercises [27]. In general, women are less fatigable than men in many isometric tasks and some dynamic tasks when young healthy men and women perform muscle contractions of similar intensity. Namely, women show a lower increase in blood lactate concentration during sprint training [28], a lower depletion of adenosine triphosphate, and a lower accumulation of its degradation products than men [29], demonstrating the central contribution of skeletal muscle metabolism to gender differences in fatigue. The anatomical differences in the structure of the musculoskeletal system between the sexes can also influence the biomechanics of postural control [30]. In addition, hormones, such as estrogen and progesterone, can influence ligament laxity and joint stability [31], and testosterone contributes to improved muscle strength in men [32]. There are not only postural differences between men and women in terms of hormonal factors, musculoskeletal structure and strength, body composition, and neuromuscular control but also as a result of different behaviors during PA. Namely, our previous findings show that young women spend more time sitting [9], engage in less PA related to sports, recreation, and leisure, and spend more time in PA related to the household and work than young men [33]. Excessive sitting and a lack of PA are usually the causes of poor posture, which typically results from an imbalance of muscles (trunk extensors and flexors) that pull on the pelvis and/or lumbar spine [34] in an attempt to maintain stability and alignment of the lumbar spine [35]. The trunk extensors are important for maintaining an upright posture and tend to become hypertonic with fatigue, exaggerating lumbar lordosis and creating anterior pelvic tilt as part of postural dysfunction [36]. In addition, the trunk flexors tend to fatigue easily and become weakened when overloaded [37]. Moreover, our previous results have shown that female students exhibited pronounced lordosis, pronounced anterior pelvic tilt, and increased trunk extensor endurance, whereas males exhibited more pronounced hyperkyphosis and increased trunk flexor endurance [9]. Both conditions, i.e., lumbar lordosis with overloaded trunk extensors and hyperkyphosis with overloaded trunk flexors, can lead to muscle fatigue [38].

Still, despite an increasing number of studies focusing on university students, there is no systematic consideration of the relationship between fatigue and a particular type and level of PA, time spent sitting, and posture between genders in university students aged 18–25 years. Thus, the aim of these analyses of part II of the cross-sectional study was to investigate the gender differences in the relationships between fatigue and PA, sleep quality, spinal curves, endurance, and balance of the trunk muscles and the possible presence of non-specific lumbar pain.

## 2. Materials and Methods

### 2.1. Participants

The present study was a cross-sectional study conducted at the University of Rijeka, Croatia, during the academic year of 2022–2025. Participants were recruited from three different faculties: the Faculty of Health Studies, the Faculty of Medicine, and the Faculty of Maritime Studies.

An estimation of the appropriate sample size for the study was guided by these two methods: (1) previous research and (2) general statistical principles. More specifically, estimation was based on the following:

(1) Earlier studies that used a similar methodology [39,40].
(2) The program MedCalc (© 2023 MedCalc Software Ltd., Ostend, Belgium), which estimated a minimum of 63 subjects needed to achieve 80% power with Cohen’s d = 0.50 for effect size, α = 0.05 type I error, and beta = 0.20 type II error [9,41].

After the research project was introduced through a public presentation, an interview was conducted with 225 volunteers. After the interview, a total of 205 volunteers met the inclusion criteria and were enrolled in the study. The inclusion criteria were healthy young adult university students aged 18 to 25 years without cardiovascular, respiratory, metabolic, autoimmune, or other systemic diseases and/or spinal pathologies, without a previous diagnosis of a systemic musculoskeletal problem or pain, and without a history of spinal or limb surgery. Individuals who used assistive devices or orthoses were also excluded from the study.

Of these 205, a total of 180 completed all required tests. The researchers obtained the necessary approvals from the Ethics Committee of the Teaching Institute of Public Health (number: 08-820-40/50-22) and the Ethics Committee of the Faculty of Health Studies of the University of Rijeka (number: 2170-1-65-23-1). Informed consent was obtained from all participants in accordance with the Declaration of Helsinki.

### 2.2. Study Design

The design of the cross-sectional study followed the STROBE Statement [42]. All measurements were carried out by the same researchers. A research protocol designed for this study consisted of three phases. The 1st phase consisted of completing the questionnaires as previously described [24]. The questionnaire for assessing fatigue was completed on the first day in order to rule out any possible additional influence of longer measurements on the degree of fatigue. The 2nd phase consisted of 2 subphases, as previously described [24], where trunk muscle endurance tests were performed. The 3rd (final) phase included the measurement of spinal curvatures. There was a 24 h break between the three main phases.

### 2.3. Outcome Measures

#### 2.3.1. Questionnaires

• Self-Reported PA and Time Spent Sitting

The IPAQ-LF was administered by trained interviewers to assess participants’ self-reported PA and sedentary behavior [43,44]. It is a reliable and valid questionnaire that health education and promotion professionals can confidently use to assess college students’ participation in PA [45]. The validity indices of the questionnaire are similar to other self-reported PA questionnaires [46]. A detailed description of the IPAQ scoring protocol, including the criteria for cutting off extreme values, is available online [43].

• Self-Reported Fatigue

Fatigue was measured with the Paediatric Quality of Life™ (PedsQL™) Multidi-mensional Fatigue Scale for young adults (age 18–25 years) (Mapi Research Trust, Lyon, France, https://eprovide.mapi-trust.org; accessed 25 October 2023) [47]. It has an excellent reliability coefficient (Cronbach’s alpha) of 0.90 and correlates significantly with the PedsQL 4.0 Generic Core Scales, which supports its construct validity [48]. It is an 18-item questionnaire consisting of the following 3 domains: (1) General Fatigue (6 items), (2) Sleep/Rest Fatigue (6 items), and (3) Cognitive Fatigue (6 items). The scale values result from the sum of the items divided by the number of answered items. A 5-point response scale is used for the self-report of young adults aged 18 to 25 years (0 = never a problem, 1 = almost never a problem, 2 = sometimes a problem, 3 = often a problem, 4 = almost always a problem). The items are reverse scored and linearly transformed into a 0–100 scale (0 = 100, 1 = 75, 2 = 50, 3 = 25, 4 = 0) so that higher scores on the PedsQL™ Multidimensional Fatigue Scale indicate a better quality of life (fewer symptoms of fatigue).

• Self-Reported Sleep Quality

SQ was measured using the Pittsburgh Sleep Quality Index (PSQI). The PSQI is a reliable and valid self-rated questionnaire that assesses SQ and disturbances over a one-month period [49,50]. The seven component scores of the PSQI have an overall reliability coefficient (Cronbach’s alpha) of 0.83 [49]. The content validity index of the PSQI is 0.91 [51]. The test distinguishes between poor and good sleep quality by measuring seven components: subjective SQ, sleep duration, sleep disturbances, use of sleep medication, and daily disturbances experienced in the past month. A score ≥ 5 indicates poor sleepers, and a score < 5 indicates people with normal SQ.

#### 2.3.2. Trunk Muscles Endurance Testing

• Trunk Extensor Endurance Testing

The trunk extensor endurance test is a reliable and valid test for assessing the muscular endurance of the torso extensor muscles that stabilize the spine (i.e., *erector spinae* and *multifidus* muscles) [52,53,54]. It is a timed test with a static, isometric contraction performed according to the modification according to McGill et al. [52]. Participants were instructed to lie on a test table in a prone position. The trunk was positioned at the level of the anterior superior iliac spine at the edge of the test table [9]. Participants kept their upper body away from the end of the table by supporting themselves with their outstretched arms on a chair directly below them. The test time was set at 180 s and measured with a stopwatch while the arms were lifted from the chair and crossed over the chest, with the hands resting on the opposite shoulders and the participants assuming the horizontal position [9]. Researcher 1 stood by the side and measured the time, and the test was terminated when participants deviated from the horizontal plane. Researcher 2 stabilized the participants’ lower body by holding the participants’ lower extremities down [54].

• Trunk Flexor Endurance Testing

A standardized trunk flexor endurance test was performed according to previously published methods [52]. The trunk flexor endurance test is a reliable and valid test that assesses the muscular endurance of the trunk flexors (i.e., *rectus abdominis*, external obliques, internal obliques, and *transversus abdominis* muscles) [53,54]. This is a timed test in which the anterior muscles are isometrically contracted to stabilize the spine until the subject shows signs of fatigue and can no longer maintain the assumed position or reach the predetermined time of 180 s. The test was performed in a supine position. Participants were in a supine position with the hips and knees flexed to 90° and the trunk resting on a wedge at a 60° angle [9]. The arms were crossed in front of the chest, and the hands were placed on the opposite shoulders. Time was measured from the moment the wedge was pushed back 10 cm until the participant re-established contact with the wedge [9]. Researcher 1 stood at the participant’s side and measured the time with a stopwatch. Stabilization of the participant’s feet was performed by researcher 2 [54].

• Balance of Trunk Muscles

The trunk extensor/flexor endurance test ratio represents a good parameter for the balance of the trunk’s musculature. It is a ratio between the endurance of the trunk extensors and the endurance of the trunk flexors. This measure is calculated from the ratio between the trunk extensor endurance and the trunk flexor endurance scores. There are no reference values for this ratio. It was modified following Kim et al. [55].

#### 2.3.3. Visual Analogue Scale (VAS) for NS-LBP

The VAS for pain was used to assess the intensity of NS-LBP in the last 4 weeks [56,57]. This is a valid and reliable test for measuring subjective characteristics or attitudes that cannot be measured directly. Here, participants indicated their level of pain by selecting the appropriate number under the picture of the facial expression and the corresponding description. The VAS for physical pain was 10 units long (0 = no pain and 10 = worst possible pain).

#### 2.3.4. Evaluation of Spinal Curvatures

Spinal curvatures (angle of thoracic kyphosis and lumbar lordosis) were measured using a non-invasive Spinal Mouse^®^ (SM) device (Idiag M360, Fehraltorf, Switzerland). This is a safe, reliable, quick, and easy-to-use method with no side effects and a suitable substitute for X-rays to measure spinal alignment and mobility, including kyphosis and lordosis [58,59,60,61,62,63,64]. It is a skin surface device that can be used in different body positions, i.e., upright standing and forward bending. The SM has acceptable metrological properties for assessing sagittal thoracic and lumbar curvature and spinal mobility. Its intraclass correlation coefficients (ICCs) for intra-rater reliability are between 0.61 and 0.96, and the ICCs for interrater reliability are between 0.70 and 0.93 [59,60,61,62,63,64].

The thoracic kyphosis and lumbar lordosis were measured while standing. The postural measurements were performed in the sagittal plane with bare feet in a relaxed standing position, i.e., in anatomical position using Idiag M360 protocol software version G6 6.4 2x. The measurements were performed in one day, and no exercises were performed before the measurement. Using the software for this device, the data displayed on the screen were used to analyze the positional relationships between each vertebra, measure the angles between the vertebrae, and calculate the angles of the spinal curvatures. The standard procedure for the upright sagittal posture was performed; the spinous process of the 7th cervical vertebra was marked as the starting point for the measurement, and the end point was marked at the level of the 3rd sacral vertebra. The posterior superior iliac spine (PSIS) was marked using an alternative method by drawing the line between the PSIS. After the line between PSIS was drawn 2 cm below the line, the position was marked with a flexible ruler. The vertical line was used to mark the center of the new line below the PSIS line so that the cross was over the S3 vertebra. The SM was placed over the C7 vertebra with the orange mark on the device over the marked starting position, and the recording was made by moving the device from top to bottom to the end point. Negative values in the lumbar curve correspond to lumbar lordosis. When assessing the thoracic spine in a standing position, values between 20° and 45° were considered neutral thoracic kyphosis, less than 20° was considered hypokyphosis, and more than 45° was considered hyperkyphosis [58]. The values of the lumbar spine were considered neutral lordosis if they ranged from 20° to 40°, below 20° was classified as hypolordosis, and more than 40° as hyperlordosis [65].

### 2.4. Statistical Analysis

The data were analyzed using *Statistica*, version 13 (TIBCO Software Inc., 2017, Palo Alto, CA, USA). The genders (independent variables) were compared through descriptive data and different domains of the PedsQL™ Multidimensional Fatigue Scale: age, body mass index (BMI) (kg/m^2^), general fatigue, sleep/rest fatigue, cognitive fatigue, and total fatigue.

Values of total fatigue were presented as interquartile ranges to obtain more precise insight into the fatigue level.

Therefore, participants were categorized into quartiles (from Q1 to Q4) of fatigue levels using the PedsQL™ Multidimensional Fatigue Scale, 19–58, 59–69, 70–79, and 80–99, where higher PedsQL™ Multidimensional Fatigue Scale scores indicate better quality of life (fewer symptoms of fatigue). Thus, Q1 includes participants with the most fatigue and Q4 with the least fatigue. Accordingly, dependent variables (percentage of each gender, level and type of PA, time spent sitting, quality of sleep, NS-LBP, angle for thoracic kyphosis and lumbar lordosis in standing position, trunk muscle endurance (trunk flexor and extensor endurance in sec), ratio of extensors/flexors of the trunk (balance) were compared between the quartiles of the same total fatigue level of both genders.

The Variance Inflation Factor (VIF) was used to determine whether multicollinearity existed in the model. All VIF values for fatigue quartiles were comfortably below 3.0 (Q1 = 1.128; Q2 = 1.224; Q3 = 1.188; Q4 = 1.309), which suggests that multicollinearity problems were not present in the model [66].

Throughout the text, the following symbols were used for fatigue quartiles: “Q1”, “Q2”, “Q3”, and “Q4”. Data distribution was tested for normality using the Kolmogorov–Smirnov test. Gender was presented as a percentage and age, BMI, fatigue, PA, time spent sitting, spine angles, and muscle endurance as the mean ± SD. The quality of sleep was presented as the median and the range. To compare dependent parametric variables (age, BMI, fatigue, PA, sitting time, spine angles, and muscle endurance) between genders, we used Student’s *t*-test, and to compare the dependent non-parametric variables quality of sleep and NS-LBP, we used the Mann–Whitney U test.

Chi-square analyses were used to examine the frequency distributions of the genders between the total fatigue quartiles.

The relationships between fatigue, PA, time spent sitting, SQ, and NS-LBP were analyzed using Pearson correlation. In the correlation analyses, the values of the correlation coefficients were considered as follows: 0.00–0.19 was considered “no relationship”, 0.20–0.39 “weak relationship”, 0.40–0.69 “medium relationship”, 0.70–0.89 “strong relationship”, and 0.90–1.00 “very strong relationship”. As the *p*-values alone do not give any indication of the size of an effect, we calculated the effect sizes for the differences between the genders as Cohen’s *d* and interpreted them as criteria: small (0.2), moderate (0.5), and large (0.8) [41]. The significance level of the statistical analyses was set at *p* < 0.05.

## 3. Results

**Figure 1:**
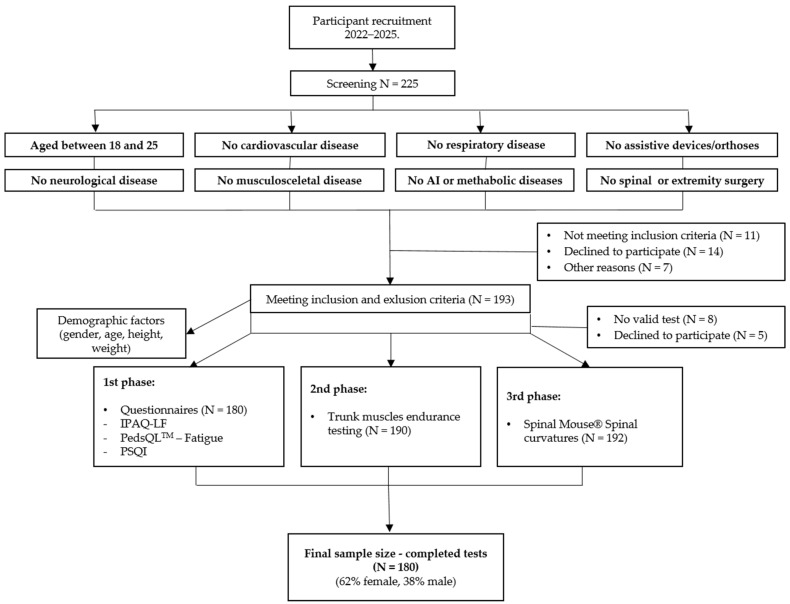
Flowchart of the number of participants at different stages in the study. AI, autoimmune; N, number; IPAQ-LF, the International Physical Activity Questionnaire-Long Form; PedsQL^TM^, Pediatric Quality of Life Inventory; PSQI, Pittsburgh Sleep Quality Index.

At baseline, a total of 225 subjects were enrolled in the study, and, after the exclusion of 45 subjects who did not meet the inclusion criteria, declined to participate, or had not completed all of the required assessments, the study was completed with 180 subjects (Figure 1). Of these, 112 (62%) were female and 68 (38%) were male.

No significant differences were found with regard to BMI and age (Table 1). The BMI values reflect the normal weight of these university students (24.8 ± 15.9). The mean age of the participants was 20.7 ± 1.9 years, and the mean BMI was 24.8 ± 15.9 kg/m^2^. Table 1 shows the descriptive data and the different domains of the PedsQL^TM^ Multidimensional Fatigue Scale for all 180 subjects. The mean ± SD of total fatigue was 68.6 ± 16.4 for female students and 67.9 ± 14.9 MET-min/wk for male students, with no statistically significant difference (*p* = 0.809; *d* = 0.04). Although female students had higher levels of general and sleep/rest fatigue, there were no statistically significant differences.

No statistically significant differences were found in the percentage of participants with total fatigue between quartiles of fatigue (*p* = 0.666) (Table 2). In general, female students’ total fatigue decreased the less they engaged in PA (from Q1 to Q4), especially during moderate or vigorous PA (Table 2). 

In addition, the Pearson correlation test revealed a statistically significant negative correlation between the scores for total fatigue and total PA in the female students, in contrast to the male students, indicating that the higher the level of PA, the greater the fatigue (Table 3).

However, this fatigue could be related to poor SQ in female students, as a negative correlation was found between the sleep/rest fatigue score and total PA during the week, suggesting that the higher the level of PA, the greater the fatigue related to sleep (Table 4).

Both genders had PSQI scores above five, indicating significant sleep difficulties. Although the male students generally had more participants with poorer sleep quality than the female students, the female students felt more fatigued when SQ was low (Q1), indicating some association between SQ and fatigue in the female students (Table 5). 

In addition, both genders showed a statistically significant negative correlation between SQ and fatigue related to sleep, indicating that the worse the students’ SQ, the greater the fatigue related to sleep (Table 6). In addition, both genders showed a statistically significant positive correlation between NS-LBP and SQ, suggesting that the presence of NS-LBP affects SQ but not sleep-related fatigue (Table 6).

Moreover, male students showed a decrease in a total fatigue during moderate and vigorous PA (from Q1 to Q4), but not during light physical activity, such as walking (Table 2). As in our previous results [9,24], male students had significantly more moderate (2031.8 ± 1831.1 vs. 1430.4 ± 1809.3, *p* = 0.009, *d* = 0.33), vigorous (3061.3 ± 2800.5 vs. 1791.4 ± 2591.3, *p* = 0.008, *d* = 0.47), and total PA (6913.3 ± 4378.1 vs. 4900.4 ± 4027.2, *p* = 0.005, *d* = 0.48) and types of PA associated with recreation, sport, and leisure (3545.9 ± 2623.4 vs. 2578.7 ± 2500.6, *p* = 0.029, *d* = 0.37) than women (Table 2). In addition, although no statistically significant difference in job-related PA was found between quartiles of fatigue, a similar pattern was found in total fatigue in female and male students, indicating that females are less fatigued when job-related PA decreases (from Q1 to Q4), which is in contrast to male students (*d* = 0.44, *d* = 0.59, *d* = 0.32, respectively) (Table 2). The similarity of the decrease in PA level through the quartiles of fatigue (from Q1 to Q4) in both genders during transport suggests that a higher level of fatigue (Q1) occurs at higher PA levels during transport (Q1) (Table 2).

For both genders, there was an increase in sitting time across fatigue quartiles (from Q4 to Q1), indicating that the more time students spent sitting, the more fatigued they were. However, female students spent significantly more time sitting than male students (6.2 ± 1.9 vs. 5.0 ± 1.6, *p* = 0.013, *d* = 0.68) (Table 2).

In addition to the already known finding [9] that significantly more male participants were hyperkyphotic (46.4 ± 9.4 vs. 42.7 ± 9.6, *p* = 0.037, *d* = 0.38), fatigue was greater with progression of hyperkyphosis (from Q4 to Q1) (Table 5), with statistically significant difference and small/medium/large effect sizes (*p* = 0.034, *d* = 0.91, *d* = 0.59, *d* = 0.29) compared to female students.

A similar finding regarding lordosis was present in the female students, i.e., they had more pronounced lordosis than the males, with a statistically significant difference (*p* = 0.009, *p* = 0.020, *p* = 0.007, *p* ≤ 0.001) and medium to large effect sizes between the sexes (*d* = 1.18, *d* = 1.65, *d* = 1.25, *d* = 0.50, *d* = 0.38), and, with more pronounced lordosis, they were more fatigued in contrast to the males (from Q4 to Q1) (Table 5).

No particular difference was found between the sexes in values related to endurance of the trunk flexors. However, the degree of fatigue, especially in men, gradually increased as the flexor endurance values increased (from Q4 to Q1) (Table 5). As previous results already showed [9], female students have significantly higher values of trunk extensor endurance (154.3 ± 34.6 vs. 138.4 ± 38.1, *p* = 0.019, *d* = 0.43), which is related to more pronounced lumbar lordosis in contrast to men, and here it can be seen that as the values increase, the degree of fatigue also increases (from Q4 to Q1), with statistically significant differences and small/large effect sizes between the sexes (*p* = 0.022, *p* = 0.016; *d* = 0.90, *d* = 1.06, *d* = 0.33, *d* = 0.22) (Table 5). As previous findings have shown [9], female students again had significantly higher ratio values and correspondingly more balanced trunk muscles (0.92 ± 0.2 vs. 0.80 ± 0.3, *p* = 0.015, *d* = 0.40) (Table 5). However, as the female students had a higher extensor/flexor ratio, fatigue was more pronounced (from Q4 to Q1) (Table 5), indicating that the trunk extensors are more fatigued when maintaining lumbar spine alignment and stability.

## 4. Strengths and Limitations

The strengths of the study are as follows. (1) The greatest strength is the complexity of the assessments, with various questionnaires and physical tests. (2) We used the most up-to-date recommended methods for this type of study. (3) We assessed total fatigue using the PedsQL™ Multidimensional Fatigue Scale, dividing the results into quartiles (levels of total fatigue) and comparing them between the genders according to dependent variables (level and type of PA, sitting time, sleep quality, spinal curvatures, the endurance and the balance of the trunk muscles, and NS-LBP). (4) We used a non-invasive device, the Spinal Mouse^®^, a safe, reliable, quick, and easy-to-use method without side effects and a suitable substitute for X-rays to measure the angular values of the spine.

The limitations of the study are as follows. (1) This includes its cross-sectional type of study. Therefore, statements regarding cause and effect cannot be made. (2) One of the main limitations is the complexity of the study due to the multiple measurements, which required participants to make extra effort to complete all tests. (3) The study included more female students. (4) In addition, we did not analyze thoracic nor cervical spine pain. (5) The study also lacked an objective measurement of PA and sedentary behavior. (6) We have not analyzed the degree of stress, cognition, dietary habits, plasma factor levels, the deficiency of which could affect fatigue, SES, or monthly fluctuation of estrogen and progesterone levels in women. (7) The device SM has its limitations, as it does not measure the cervical part of the spine.

## 5. Discussion

In our study examining the relationship between fatigue and a certain level and type of PA, sedentary behavior, SQ, NS-LBP, and posture between genders, female students were found to have greater total fatigue when they engaged in more PA in general (walking, moderate, vigorous), in contrast to males, who had greater total fatigue when they engaged in less moderate and less vigorous PA. A similar pattern emerged in female students for certain types of PA, i.e., job-related PA, PA during transport, housework and recreation, and sport and leisure. Moreover, female students showed more sleep/rest-related fatigue as they had more total PA during the week. Male students showed more pronounced fatigue when they had less job-related PA and less PA during recreation, sport, and leisure. With increasing sedentary behavior, total fatigue was more pronounced in both genders, although female students spent significantly more of their daily time sitting them males. Poorer SQ correlated with NS-LBP and higher levels of sleep-related fatigue in female students. In addition, male students became more fatigued when they had more pronounced hypekyphosis, whereas female students became more fatigued when they had more pronounced lordosis. Furthermore, fatigue was more pronounced in female students as they had a higher value of the extensor/flexor ratio, suggesting that the trunk extensors are more fatigued in maintaining alignment and stability of the lumbar spine.

### 5.1. Relationship Between Fatigue, PA, Time Spent Sitting, and SQ in University Students

The assessment of fatigue is a complex process, as it involves the measurement of different dimensions of life with emotional, behavioral, and cognitive components. According to many studies, PA is a healthy behavior that provides good combat against the feeling of fatigue [67] and for sedentary behavior characterized by prolonged inactivity, leading to muscle weakness, poor cardiovascular health, and fatigue [68]. However, in our study, the relationship between fatigue and PA differs between genders when considering the different types of PA measured during a week in the academic population, i.e., university students. Namely, along with academic stress, females showed more fatigue during the week after performing more PA in general, which is completely opposite to the results in males. Moreover, they have more job-related PA and more PA related to housework and engaged in less PA during recreation, sport, and leisure, which, all together, could be a reason for some kind of burnout [69,70]. Namely, women are more likely to be affected by burnout, a state of emotional, physical, and mental exhaustion. This situation and the feeling of fatigue worsen when they spend significantly more time sitting during the week. Interrupting prolonged sitting with moderate PA could also have a positive effect on cognitive function [71] by increasing intracerebral blood flow [72]. However, in our study, there was no association between PA and cognitive fatigue. In female students, the most significant association between total PA and fatigue was related to sleep/rest. In addition, females showed that their poorer SQ was associated with greater fatigue. PA, especially when it is moderate and performed as a leisure activity rather than strenuous work-related PA, can improve SQ, and high levels of physical exertion at work can negatively affect sleep [73]. Researchers suggest that both sleep and exercise are critical for the “prevention“ of somatic symptoms [74]. In addition, our previous findings related to the measurement of serum vitamin D levels showed that students with vitamin D insufficiency showed more fatigue, especially related to sleep/rest, higher levels of psychological distress, and a greater frequency of headaches [75]. However, we have not analyzed any difference between the sexes. Thus, fatigue and academic stress can be also associated with an unhealthy diet [76]. In addition, socioeconomic status (SES) can have a major impact on overall well-being and possible fatigue. SES can be closely linked not only to regular PA but also to various areas of quality of life and health. Namely, individuals with higher SES often exhibit higher levels of PA and experience a better quality of life [77] than those with lower SES, who may have less access to resources that promote well-being, such as healthy food, quality healthcare, and a safe living environment, which exacerbate fatigue. Fatigue can have a negative impact on mental health, leading to conditions like depression and anxiety, which in turn affects socioeconomic outcomes. In addition, individuals with lower income and education levels may experience higher levels of stress due to financial instability and limited opportunities, which in turn may contribute to fatigue [78]. In essence, socioeconomic factors can significantly influence SQ, with lower SES often associated with poorer sleep and increased fatigue due to the complex interplay of stress, lifestyle, and neighborhood factors [79,80]. Furthermore, the potential impact of fluctuations in estrogen and progesterone levels during the menstrual cycle can also have a significant impact on energy levels and cause sleep disturbances that contribute to fatigue in women [81]. In our study, we did not analyze the association between fatigue and dietary habits, SES, cognition, psychological stress, deficiency of certain plasma factors, such as iron or vitamin D, especially in women, or monthly fluctuation of estrogen and progesterone levels, but this will be included in our future investigations.

### 5.2. Relationship Between Fatigue, Posture, NS-LBP, and SQ in University Students

Moreover, in our previous study, we showed that female students, in contrast to male students [9], exhibit other postural abnormalities, including more pronounced lordosis, anterior pelvic tilt, and greater endurance of the trunk extensors, which are also most likely associated with the presence of concomitant NS-LBP. Now, we have confirmed that although female students spend more time sitting, they may also show more fatigue because of this. This behavior leads to significant postural adjustment and causes fatigue of certain trunk muscles in maintaining stability of the lumbar spine [35]. In contrast to the females, the male students exhibited hyperkyphosis and increased endurance of the trunk flexors. Not only poor posture but also the stress of intense physical training can increase the risk of developing hyperkyphosis in certain sports [82,83]. Both conditions, i.e., lumbar lordosis with overloaded trunk extensors and hyperkyphosis with overloaded trunk flexors, can lead to muscle fatigue, defined as a decrease in power output with equal or higher muscle activation, which is one of the causes of an unregulated postural control system and a possible predisposing factor for NS-LBP [38]. Muscle fatigue disrupts postural control through its negative effects on the accuracy of sensory information, the integration of peripheral afferents into the central nervous system, and the effectiveness of motor commands [84].

Additionally, NS-LBP is a constant drain on energy because you have to constantly deal with the pain and therefore cannot sleep well. An imbalance of core muscles and a less active lifestyle can make this fatigue worse [85]. NS-LBP affects several areas of quality of life, not only SQ, and also causes fatigue [38]. In our study, NS-LBP correlated with SQ in both genders, especially in female students. In addition, sleep deprivation has been shown to lead to hyperalgesia and decreased pain thresholds and cognitive abilities to cope with pain. Therefore, it is not surprising that sleep problems are associated with poor performance on both physical and cognitive tasks, including decreased job and/or academic performance [38]. In our study, we did not actually measure what performance female students had, but, based on our results, we only can assume that females are more fatigued when having more job-related PA. Aside from the fact that women prefer more sedentary activities [86], numerous research findings suggest that female students experience higher levels of stress compared to men [87], which may have an additional impact on overall fatigue levels. In addition, women experience higher levels of work-related stress compared to men. This is due to a combination of factors, including societal expectations, workplace dynamics, and the pressure to balance work and family life [88]. However, a question remains: Why does PA during work or housework combined with less PA during recreation, sport, and leisure time in female students lead to being more fatigued than men during study time? This area should be researched in greater detail.

## 6. Conclusions

Based on the results obtained, we can assume that fatigue is negatively associated with PA in female students and positively associated with PA in male students. In addition, postural deformities, the presence of NS-LBP, and lower SQ increase fatigue in both genders. However, why exactly PA during work or housework in combination with less PA during recreation, sport, and leisure time in female students leads them to be more fatigued than men during study time should be further confirmed and investigated in more detail. University student health promotion requires gender-specific PA and postural interventions, because these approaches address the unique physiological and behavioral needs of young men and women to improve musculoskeletal health and minimize injury while promoting long-term physical well-being. Educational programs that adapt to these differences lead to more balanced and sustainable health outcomes in educational settings.

## Figures and Tables

**Table 1:** Demographic characteristics and gender differences in different fatigue domains of the PedsQL^TM^ Multidimensional Fatigue Scale (N = 180).

Variable	Females	Males	Total	*p* Value	d
Gender, n (%)	112 (62)	68 (38)	180 (100)	NA	NA
Age, mean ± SD	20.7 ± 1.7	20.9 ± 1.8	20.7 ± 1.9	0.241	0.11
BMI (kg/m^2^), mean ± SD	24.1 ± 2.8	24.5 ± 3.2	24.8 ± 15.9	0.621	0.13
General fatigue	70.3 ± 19.4	67.4 ± 17.9	68.9 ± 17.9	0.350	0.15
Sleep/rest fatigue	64.2 ± 16.5	63.0 ± 16.8	63.4 ± 16.7	0.668	0.07
Cognitive fatigue	71.5 ± 23.1	73.6 ± 18.7	72.8 ± 20.4	0.548	0.09
Total fatigue	68.6 ± 16.4	67.9 ± 14.9	68.2 ± 15.4	0.809	0.04

NA, not applicable; n, number; SD, standard deviation; BMI, body mass index. Statistical analysis: Student’s *t*-test; *p* < 0.05; *d*, Cohen’s value for effect size.

**Table 2:** Comparison of IPAQ-LF domains (intensity and type of PA, including time spent sitting) between genders according to the quartiles of self-reported fatigue.

Variable (Per Fatigue Quartiles/MET-min/wk)	Females	Males	Total	*p* Value	*d*
Q1 (19–58)					
Q2 (59–69)					
Q3 (70–79)					
Q4 (80–99)					
^a^ Participants, n (%)				(*df* = 3) 0.666	NA
Q1	30/46 (65.2)	16/46 (34.8)	46/180 (25.6)		
Q2	26/42 (61.9)	16/42 (38.1)	42/180 (23.3)		
Q3	30/46 (65.2)	16/46 (34.8)	46/180 (25.6)		
Q4	26/46 (56.5)	20/46 (43.5)	46/180 (25.6)		
**Level of PA**					
^b^ Walking (MET-min/wk), mean ± SD			2249.4 ± 2049.0		
Q1	2681.5 ± 2520.8	2773.5 ± 2787.7		0.922	0.03
Q2	1390.4 ± 1462.0	2334.0 ± 1705.1		0.108	0.59
Q3	2206.4 ± 1287.0	2689.5 ± 3152.9		0.509	0.2
Q4	2101.0 ± 1749.2	1979.4 ± 1553.8		0.827	0.07
Total	2193.0 ± 1949.4	2378.5 ± 2274.2		0.577	0.08
^b^ Moderate (MET-min/wk), mean ± SD			1515.5 ± 1693.5		
Q1	1967.8 ± 2511.8	1358.3 ± 746.4		0.459	0.33
Q2	1177.9 ± 1574.1	1799.5 ± 1276.3		0.265	0.43
Q3	1666.7 ± 1737.5	2207.9 ± 1874.5		0.392	0.2
Q4	875.5 ± 798.6	1831.3 ± 1668.8		0.027 *	0.73
Total	1430.4 ± 1809.3	2031.8 ± 1831.1		0.009 *	0.33
^b^ Vigorous (MET-min/wk), mean ± SD			2159.9 ± 2699.6		
Q1	2067.9 ± 2953.3	2431.1 ± 2198.9		0.739	0.14
Q2	1454.0 ± 2039.0	1997.8 ± 1439.2		0.452	0.3
Q3	2543.2 ± 3389.6	3867.8 ± 3876.6		0.339	0.36
Q4	884.8 ± 937.2	3669.1 ± 3113.4		<0.001 *	1.21
Total	1791.4 ± 2591.3	3061.3 ± 2800.5		0.008 *	0.47
^b^ Total PA (MET-min/wk), mean ± SD			5839.1 ± 4559.7		
Q1	6562.1 ± 6039.1	5537.6 ± 3852.1		0.594	0.2
Q2	4088.4 ± 3713.9	5454.4 ± 3366.7		0.298	0.38
Q3	6416.3 ± 4570.1	7865.8 ± 6065.3		0.422	0.27
Q4	3861.3 ± 2331.8	7532.8 ± 4569.6		0.003 *	1.01
Total	4900.4 ± 4027.2	6913.3 ± 4378.1		0.005 *	0.48
**Type of PA**					
^b^ Job-related PA (MET-min/wk), mean ± SD			1044.4 ± 2037.9		
Q1	1910.5 ± 4524.2	450.0 ± 1064.2		0.28	0.44
Q2	496.4 ± 1752.8	526.3 ± 1038.9		0.957	0.02
Q3	460.3 ± 1474.3	2177.2 ± 3773.9		0.053	0.59
Q4	1208.1 ± 2739.9	2246.2 ± 3663.1		0.293	0.32
Total	921.7 ± 2785.6	1252.6 ± 2647.3		0.473	0.12
^b^ Transportation (MET-min/wk), mean ± SD			986.5 ± 985.1		
Q1	1128.6 ± 880.1	1406.0 ± 1817.7		0.532	0.19
Q2	664.5 ± 766.9	1042.1 ± 923.8		0.222	0.44
Q3	1092.9 ± 760.9	1078.0 ± 1526.7		0.968	0.01
Q4	785.5 ± 683.6	887.0 ± 829.6		0.669	0.13
Total	924.9 ± 788.5	1082.7 ± 1249.4		0.341	0.15
^b^ Housework (MET-min/wk), mean ± SD			913.4 ± 1218.4		
Q1	946.3 ± 1115.6	866.4 ± 1219.1		0.836	0.06
Q2	783.3 ± 1115.9	1390.2 ± 1455.9		0.188	0.46
Q3	1451.4 ± 1895.9	628.2 ± 1895.9		0.148	0.43
Q4	426.9 ± 453.7	803.2 ± 871.0		0.09	0.54
Total	907.4 ± 1312.4	909.7 ± 1035.2		0.991	0
^b^ Recreation, sport, and leisure -time (MET-min/wk), mean ± SD			2872.3 ± 2548.6		
Q1	2555.1 ± 2542.1	3193.6 ± 1539.2		0.461	0.3
Q2	2086.8 ± 2219.2	2948.7 ± 1972.6		0.295	0.41
Q3	3456.4 ± 3041.3	3347.8 ± 2348.9		0.914	0.03
Q4	1792.9 ± 1446.2	4432.8 ± 3494.6		0.003 *	0.98
Total	2578.7 ± 2500.6	3543.9 ± 2623.4		0.029 *	0.37
^b^ Sitting time (MET-min/wk), mean ± SD			5.7 ± 2.0		
Q1	6.3 ± 1.8	5.8 ± 3.3		0.601	0.18
Q2	6.1 ± 1.9	5.4 ± 2.2		0.371	0.34
Q3	5.7 ± 1.3	4.6 ± 2.3		0.092	0.58
Q4	5.9 ± 1.4	5.1 ± 1.2		0.089	0.61
Total	6.2 ± 1.9	5.0 ± 1.6		0.013 *	0.68

Key findings: In general, female students’ total fatigue decreased the less they engaged in PA (from Q1 to Q4), especially during moderate or vigorous PA. In contrast, male students showed a decrease in total fatigue during moderate and vigorous PA (from Q1 to Q4). Male students had significantly more moderate, vigorous, and total PA and types of PA associated with recreation, sport, and leisure than women. There was an increase in sitting time across fatigue quartiles (from Q4 to Q1) in both genders, indicating that the more time students spent sitting, the more fatigued they were. Female students spent significantly more time sitting than male students. Statistical analysis and abbreviations: ^a^ Chi-square test; ^b^ Student’s *t*-test; * statistical significance < 0.05; *d*, Cohen’s value for effect size; n, number; NA, not applicable; PA, physical activity; MET, metabolic equivalent of task; wk, week; Q, quartile; *df*, degree of freedom for error; SD, standard deviation.

**Table 3:** Pearson correlation analyses between total fatigue and certain types or levels of PA.

Variable	Total Fatigue (PedsQL™ Multidimensional Fatigue Scale)			
	Female		Male	
	*r*	*p*	*r*	*p*
Type of PA (IPAQ-LF, MET-min/wk)				
Job-related PA (MET-min/wk)	−0.104	0.312	0.192	0.159
Transportation (MET-min/wk)	−0.152	0.140	0.167	0.223
Housework (MET-min/wk)	−0.081	0.435	−0.043	0.751
Recreation, sport, and leisure time (MET-min/wk)	−0.104	0.311	0.187	0.181
Sitting time (h/day)	0.027	0.800	−0.097	0.692
Level of PA (IPAQ-LF, MET-min/wk)				
Walking (MET-min/wk)	−0.153	0.136	−0.005	0.689
Moderate (MET-min/wk)	−0.153	0.136	0.158	0.248
Vigorous (MET-min/wk)	−0.090	0.385	0.184	0.215
Total (MET-min/wk)	−0.230	0.028 *	0.158	0.257

Key findings: Female students were found to have a statistically significant negative correlation between total fatigue scores and total PA, in contrast to male students, suggesting that the higher the level of PA, the greater the fatigue. Statistical analysis and abbreviations: *r*, Pearson correlation coefficient; * *p* < 0.05; PedsQL™, Pediatric Quality of Life; PA, physical activity; MET, metabolic equivalent of task; wk, week; h, hour; IPAQ-LF, International Physical Activity Questionnaire-Long Form.

**Table 4:** Pearson correlation analyses between total PA and different fatigue domains.

Variable	Total PA (IPAQ-LF, MET-min/wk)			
	Female		Male	
PedsQL™ Multidimensional Fatigue Scale Domains	*r*	*p*	*r*	*p*
General fatigue	−0.161	0.127	0.187	0.178
Sleep/rest fatigue	−0.236	0.024 *	0.151	0.279
Cognitive fatigue	−0.186	0.077	0.071	0.609

**Key findings:** A statistically significant negative correlation was found between sleep/rest fatigue score and total PA in female students during the week, suggesting that a higher level of PA leads to more fatigue related to sleep. **Statistical analysis and abbreviations:** *r*, Pearson correlation coefficient; * *p* < 0.05; PA, physical activity; MET, metabolic equivalent of task; wk, week; PedsQL™, Pediatric Quality of Life; IPAQ-LF, International Physical Activity Questionnaire-Long Form.

**Table 5:** Comparison of SQ, NS-LBP, spinal curvatures, and endurance and balance of the trunk muscles between genders according to the quartiles of self-reported fatigue.

Variable (Per Fatigue Quartiles/MET-min/wk)	Females	Males	Total	*p* Value	*d*
Q1 (19–58)					
Q2 (59–69)					
Q3 (70–79)					
Q4 (80–99)					
^a^ Sleep quality, median (range)			6 (0–17)		NA
Q1	7 (3–10)	6 (2–17)		0.379	
Q2	5 (2–7)	6 (2–12)		0.728	
Q3	5 (2–9)	4 (2–11)		0.618	
Q4	5 (2–11)	6.5 (2–12)		0.303	
Total	6 (2–11)	6 (2–17)		0.723	
^a^ NS-LBP, median (range)			2 (0–8)		NA
Q1	2 (0–8)	2 (0–8)		0.346	
Q2	2 (0–7)	1 (0–6)		0.248	
Q3	1 (0- )	2 (0–6)		0.733	
Q4	1 (0–6)	2 (0–4)		0.557	
Total	2 (0–8)	2 (0–8)		0.21	
^b^ Kyphosis straight standing (°), mean ± SD			44.2 ± 9.7		
Q1	40.3 ± 8.0	49.0 ± 10.8		0.034 *	0.91
Q2	41.8 ± 9.4	46.8 ± 7.3		0.143	0.59
Q3	43.9 ± 8.0	46.5 ± 9.8		0.443	0.29
Q4	44.3 ± 12.9	45.0 ± 10.0		0.861	0.06
Total	42.7 ± 9.6	46.4 ± 9.4		0.037 *	0.38
^b^ Lordosis straight standing (°), mean ± SD			−30.9 ± 9.4		
Q1	−35.3 ± 7.0	−26.3 ± 8.1		0.009 *	1.18
Q2	−36.4 ± 9.3	−28.1 ± 8.3		0.020 *	1.65
Q3	−36.4 ± 10.7	−24.7 ± 7.7		0.007 *	1.25
Q4	−31.4 ± 8.5	−27.1 ± 8.5		0.188	0.5
Total	−34.2 ± 8.9	−26.3 ± 7.9		<0.001 *	0.93
^b^ Trunk flexor endurance, mean ± SD			167.9 ± 32.2		
Q1	166.9 ± 32.1	178.7 ± 4.6		0.222	0.51
Q2	177.2 ± 10.8	176.0 ± 12.0		0.803	0.1
Q3	166.2 ± 29.9	160.8 ± 35.4		0.43	0.16
Q4	170.8 ± 25.3	159.5 ± 48.6		0.43	0.29
Total	170.0 ± 26.1	164.8 ± 39.4		0.645	0.15
^b^ Trunk extensor endurance, mean ± SD			147.9 ± 36.8		
Q1	170.6 ± 21.9	145.4 ± 32.7		0.022 *	0.9
Q2	153.0 ± 33.2	113.7 ± 40.0		0.016 *	1.06
Q3	149.4 ± 35.3	136.2 ± 42.9		0.35	0.33
Q4	145.0 ± 41.1	153.4 ± 33.5		0.571	0.22
Total	154.3 ± 34.6	138.4 ± 38.1		0.019 *	0.43
^b^ Extensors/flexors ratio (balance), mean ± SD			0.92 ± 0.5		
Q1	1.01 ± 0.2	0.88 ± 0.2		0.075	0.65
Q2	0.87 ± 0.3	0.64 ± 0.3		0.017 *	0.76
Q3	0.93 ± 0.3	0.86 ± 0.3		0.555	0.23
Q4	0.88 ± 0.2	0.78 ± 0.3		0.297	0.39
Total	0.92 ± 0.3	0.80 ± 0.3		0.015 *	0.4

**Key findings:** Male students had, in general, more participants with poorer SQ, but the female students felt more fatigued when SQ was low (Q1), indicating some association between SQ and fatigue in the female students. Fatigue was greater in male students with progression of hyperkyphosis (from Q4 to Q1), whereas it was greater in female students with more pronounced lordosis (from Q4 to Q1). The degree of fatigue, especially in men, gradually increased as the flexor endurance values increased (from Q4 to Q1). As the values of trunk endurance in females increased, the degree of fatigue also increased (from Q4 to Q1). Female students showed significantly higher ratio values and, correspondingly, more balanced trunk muscles than males. However, as the female students had a higher extensor/flexor ratio, fatigue was more pronounced (from Q4 to Q1), indicating that the trunk extensors are more fatigued when maintaining lumbar spine alignment and stability. **Statistical analysis and abbreviations:** ^a^ Mann–Whitney U test; ^b^ Student’s *t*-test; * statistical significance < 0.05; *d*, Cohen’s value for effect size; NA, not applicable; wk, week; Q, quartile; *df*, degree of freedom for error; SD, standard deviation; NS-LBP, non-specific low back pain; SQ, sleep quality.

**Table 6:** Pearson correlation analyses between SQ, NS-LBP, and fatigue related to sleep/rest.

Variable	Sleep/Rest Fatigue (PedsQL™ Multidimensional Fatigue Scale)				PSQI (SQ)			
	Female		Male		Female		Male	
	*r*	*p*	*r*	*p*	*r*	*p*	*r*	*p*
**PSQI (SQ)**	−0.237	0.021 *	−0.311	0.023 *	/	/	/	/
**VAS (NS-LBP)**	−0.042	0.689	−0.107	0.437	0.314	0.002 **	0.316	0.021 *

**Key findings:** Both genders showed a statistically significant negative correlation between SQ and fatigue related to sleep, indicating that the worse the students’ SQ, the greater the fatigue related to sleep. In addition, both genders showed a statistically significant positive correlation between NS-LBP and SQ, suggesting that the presence of NS-LBP affects SQ. **Statistical analysis and abbreviations:** *r*, Pearson correlation coefficient; * *p* < 0.05 and ** *p* < 0.01; PSQI, Pittsburgh Sleep Quality Index; VAS, Visual Analogue Scale; NS-LBP, non-specific low back pain; PedsQL™, Pediatric Quality of Life.

## Data Availability

Data supporting this article are available from the corresponding author upon reasonable request.

## Abbreviations

The following abbreviations are used in this manuscript:

PA	Physical activity
IPAQ-LF	International Physical Activity Questionnaire-Long Form
PedsQL	Pediatric Quality of Life
SQ	Sleep quality
NS-LPB	Non-specific low back pain
STROBE	Strengthening the Reporting of Observational Studies in Epidemiology
PSQI	Pittsburgh Sleep Quality
VAS	Visual Analogue Scale
SM	Spinal Mouse
ICC	Interclass Correlation Coefficient
PSIS	Posterior superior iliac spine
BMI	Body mass index
MET	Metabolic Equivalent of Tasks
NA	Not applicable
df	Degree of freedom
SD	Standard deviation
SES	Socioeconomic status
